# Exploring Methods of Adjusting VO_2_max for Body Size to Estimate Aerobic Capacity in People Presenting for Metabolic and Bariatric Surgery

**DOI:** 10.1002/osp4.70105

**Published:** 2025-12-22

**Authors:** Rebecca Dostan, Sara Slayman, Belinda Durey, Brett Tarca, Justin Bessell, Kade Davison

**Affiliations:** ^1^ Alliance for Research in Exercise Nutrition and Activity (ARENA) Allied Health and Human Performance University of South Australia Adelaide South Australia Australia; ^2^ Australian Metabolic and Obesity Surgery Adelaide South Australia Australia; ^3^ School of Health Science University of New South Wales Sydney New South Wales Australia

**Keywords:** metabolic and bariatric surgery, obesity, oxygen consumption, perioperative care

## Abstract

**Background:**

In perioperative settings exercise testing can be used to assess a patient's physical fitness, with VO_2_max used as a marker to indicate fitness and subsequent risk of adverse surgical outcomes. However, the commonly used reporting methods of VO_2_max may be problematic in populations with excessive FM such as those awaiting MBS for obesity. Hence, alternative ways of expressing VO_2_max will be explored.

**Methods:**

Historical data from individuals presenting for MBS were analyzed. Predicted VO_2_max values were adjusted relative to the participants measured and “normative” body mass, where each prediction was assigned a classification score. Predicted VO_2_max adjusted to the participants measured FFM was also considered. Data were compared to individuals that are lean and sedentary as well as those with obesity from a previous study.

**Results:**

Data from 20 participants awaiting MBS (43.15 ± 11.82 years, 68.50 ± 8.39 cm, 115.29 ± 16.82 kg and 59.93 ± 8.69 kg FFM) were examined. Predicted VO_2_max relative: to total mass was 20.15 ± 5.00 mL/kg/min; normative body mass was 30.04 ± 6.58 mL/kg/min; and FFM was 38.55 ± 9.08 mL/kg FFM/min. Median fitness classifications increased from very poor to poor when predicted VO_2_max was expressed relative to normative body mass.

**Conclusions:**

Reporting predicted VO_2_max results relative to body mass may be underestimating fitness and overestimating surgical risk in people who are very obese.

## 1 Introduction

Prior to undergoing metabolic and bariatric surgery (MBS), extensive health screening is completed by patients to ensure they are fit for surgery and weight loss can be sustained post‐surgery [1, 2]. As a part of this process, the functional capacity of the cardiorespiratory system can be assessed by exercise testing. When functional capacity is being assessed, the maximal rate of oxygen consumption (VO_2_max) is used [3]. Therefore, VO_2_max can be used to assist in the evaluation of surgical risk and subsequent risk of adverse outcomes, with lower values inferring greater risk. Particularly in the case of individuals that are frail, VO_2_max can be a good indicator of surgical risk [4]. These adverse outcomes resultant from the stresses of surgery can include arrythmia's, cardiac arrest, gastrointestinal bleeding, pneumonia, and stroke [5]. However, the relationship between VO_2_max and surgical risk becomes less clear in the typically younger and more functional demographic undergoing weight loss surgery. As regular physical activity is an important aspect of weight loss maintenance, exercise testing and fitness assessment pre‐surgery is also considered an important aspect of capacity to adopt the ideal health behaviors post‐surgery [6].

When maximal oxygen consumption results are reported, they are frequently expressed in absolute and relative terms [7]. Absolute VO_2_max is the maximal amount of oxygen consumed by all tissues and is expressed as L/min. In comparison, relative VO_2_max aims to account for total tissue volume so is the maximal amount of oxygen consumed relative to an individual's body mass and is expressed as mL/kg/min [8]. However, there are known limitations to both these measures of reporting.

VO_2_max expressed in absolute terms does not take into consideration the expected differences due to physical size of an individual, with ultimately a larger heart and volume of working muscle generating a larger VO_2_. Whilst VO_2_max expressed relative to total body mass (mL/kg/min) accounts for these factors and is the typical way for individuals or groups to be compared, this approach does not differentiate between the composition of total body mass (e.g., fat mass [FM] and fat free mass [FFM]).

The lack of differentiation within body composition may be problematic in people with excessive FM, such as those awaiting MBS for obesity. Body size and active tissue (primarily muscle) need to be accounted for as active tissue uses more oxygen and requires more energy [9]. However, when there is excessive FM, the proportion between body mass and VO_2_ demand may not be proportional, potentially leading to distorted results. Therefore, factors including body mass and body composition are likely to influence VO_2_max values, independent of physical fitness, being the ability of the cardiorespiratory, endocrine, and neuromuscular systems to deliver and extract oxygen to perform physical work [10].

Whilst the reporting of standalone VO_2_max (commonly expressed in relative terms) can assist with the evaluation of surgical risk, the result is often compared to either a known threshold or to normative data for both age and gender. These comparisons are typically performed to provide greater context to the reported VO_2_max result. However, problems currently exist with both these methods of comparison for people that are presenting with excessive fat mass, such as those presenting for MBS.

VO_2_max thresholds for adverse surgical outcomes are frequently determined by studies that are often based on elderly populations and have normal or low body mass index (BMI), which is the opposite to that of populations considered for MBS [11, 12]. In addition, whilst the VO_2_max result, when compared to normative data, allows the result to be classified on percentile rankings, the normative data and subsequent classifications are frequently developed from samples that are not representative of individuals with very high proportions of FM [13].

Currently, there is limited research exploring different approaches for normalizing VO_2_max in individuals with proportionally high FM and those awaiting MBS. Therefore, this study will aim to explore different representations of VO_2_max and how these might influence classification and decision making. Specifically, the study aimed to review data from a selection of individuals who underwent aerobic fitness and body composition testing as part of their assessment for suitability for MBS for weight loss. This analysis was opportunistic in nature and aimed to explore an emerging phenomenon as a potential call to others to undertake prospectively designed analyses to further understand the issues presented here. Specifically, this study aimed to examine how reporting predicted VO_2_max relative to measured body mass (mL/kg/min), normative body mass (mL/kg/min), and FFM (mL/kg FFM/min) influence the expression of VO_2_max results in people who are awaiting MBS.

## 2 Methods

### 2.1 Study Design and Population

Data for individuals who underwent MBS between January 2017 and June 2017 were collected from the Australian Metabolic and Obesity Surgery Clinic (Adelaide, South Australia). This included gender, age at time of surgery, height (cm), weight (kg), BMI (kg/m^2^), predicted VO_2_max (mL/kg/min), FFM (kg) and, body fat (BF) (%). Those included were (1) presenting for MBS at the Australian Metabolic and Obesity Surgery Clinic in 2017, (2) adults aged between 18 and 64 inclusive, and (3) BMI ≥ 30 kg/m^2^. Consent was previously established by the participants to utilize their data. Ethical approval from the University of South Australia Human Research Ethics Committee was provided for use of de‐identified patient records for this analysis (Protocol No. 204467).

### 2.2 Primary Outcomes

Predicted VO_2_max was the primary measure of interest that was calculated via the Astrand rhyming protocol [14]. This protocol required the participant to cycle at a resistance that elicits a steady state heart rate between 125 and 170 beats per minute (bpm) over a 5 min period. The participants heart rate (bpm) and watts were recorded each minute. If the heart rate at minutes five and six were not within five beats/min, the participant was asked to cycle for an additional minute. Predicted VO_2_max (L/min) were calculated by inputting steady state heart rate (bpm) and workload adjusted to kp/m/min into the equation (0.00212 × workload (kp/m/min) + 0.299)/(0.769 × HRmax − 48.5) × 100 for males and (0.00193 × workload (kp/m/min) + 0.326)/(0.769 × HRmax − 56.1) × 100 for females [14]. A prediction of the participants relative VO_2_max was then determined by multiplying by 1000 and dividing by weight (kg). Body composition estimates were obtained via InBody 270 bioelectrical impedance (BIA) scales utilizing the manufactures protocol (Biospace, California, USA).

### 2.3 Adjustment for Normative Body Mass

The participants prediction of VO_2_max in L/min was adjusted to correct for population normative body mass. Body mass index datum collected by the Australian Bureau of Statistics National Healthy Survey was used as a reference for normative BMI (kg/m^2^) data for age and gender within an Australian population [15]. To calculate normative body mass the participants height (cm) and normative BMI (kg/m^2^) was imputed into the following equation, $\text{normative}\;\text{BMI}\times {\left(\frac{\text{height}}{100}\right)}^{2}$. Using the adjusted normative body mass and original prediction of VO_2_max, an adjusted VO_2_max for population normative body mass was calculated (mL/kg/min).

The original prediction of VO_2_max (mL/kg/min) and the predicted VO_2_max adjusted for population normative body mass results were categorized using the American College of Sports Medicine (ACSM) Fitness Categories for Maximal Aerobic Power for Men and Women by Age [16]. Each VO_2_max classification was assigned a classification score (Very Poor = 1.00, Poor = 2.00, Fair = 3.00, Good = 4.00, Excellent = 5.00, Superior = 6.00).

### 2.4 Adjustment for FFM

The participants body compositions including their FFM were assessed via InBody 270 BIA scales (Biospace, California, USA). The participant's measured weight was adjusted to include only their FFM (kg). Using the adjusted weight for FFM and the original predicted VO_2_max, an adjusted VO_2_max for FFM was calculated (mL/kg FFM/min) by multiplying the original prediction of VO_2_max by 1000 and dividing by the participants FFM.

### 2.5 Data Analysis

Unpaired *t*‐tests were employed to compare the demographic, anthropometric and predicted VO_2_max data between the male and female participants. The unpaired *t*‐tests were calculated under the hypothesis that ${µ}_{1}\neq {µ}_{2}$, using the following equation $t=\frac{\bar{x_{1}}-\bar{x_{2}}}{s_{p}\sqrt{\frac{1}{n_{1}}+\frac{1}{n_{2}}}}$, where $\bar{x_{1}}$ is the mean of sample 1, $\bar{x_{2}}$ is the mean of sample 2, $n_{1}$ is the sample size of sample 1, $n_{2}$ is the sample size of sample 2 and $s_{p}$ is the pooled sample deviation. The tests were performed under the assumption that the samples are independent, approximately normally distributed and of equal variance [17]. However, paired *t*‐tests were employed to compare the results between the predictions of VO_2_max, with VO_2_max expressed relative to total mass (mL/kg/min) used as the reference group. The paired *t*‐tests were performed under the assumption that the dependent variable is continuous, independent, approximately normally distributed and does not contain any outliers.

## 3 Results

Data from 17 females and three males awaiting MBS were extracted from records for analysis. Participant demographic, anthropometric and VO_2_max data can be seen in Table 1. Mean height, FFM and predicted absolute VO_2_max were significantly greater in the male participants when compared to their female counterparts. However, mean BF% is significantly greater within the female participants.

**TABLE 1:** Comparative characteristics of the male and female participants who underwent metabolic and bariatric surgery in 2017.

Variable	All participants (*n* = 20)	Men (*n* = 3)	Women (*n* = 17)
Age (years)	43.15 ± 11.82	45.67 ± 13.58	42.71 ± 11.90
Height (cm)	168.50 ± 8.39	180.33 ± 6.03	166.41 ± 6.94^b^
Body mass (kg)	115.29 ± 16.82	130.67 ± 25.01	112.58 ± 14.34
Measured BMI (kg/m^2^)	40.58 ± 4.90	40.07 ± 6.66	40.67 ± 4.78
FFM (kg)	59.93 ± 8.69	74.40 ± 9.81	57.37 ± 5.61^b^
BF (%)	47.25 ± 4.78	40.03 ± 0.46	48.52 ± 3.95^b^
Predicted absolute VO_2_max (L/min)	2.31 ± 0.62	3.07 ± 0.13	2.17 ± 0.57^a^
Body Mass adjusted for age normative BMI (kg)^c^	76.38 ± 9.49	94.02 ± 3.96	73.27 ± 6.03^b^

*Note:* Data presented as Mean ± standard deviation.

Abbreviations: BF, body fat; BMI, body mass index; FFM, fat free mass.

^a^ *p* = < 0.05 (between Males and Females).

^b^ *p* = < 0.01 (between Males and Females).

^c^ Atypical anthropometric variable.

VO_2_max expressed relative to FFM is consistently greater across all groups, when compared to predicted VO_2_max relative to total mass and VO_2_max for population normative adjusted body mass (Table 2).

**TABLE 2:** Predicted VO_2_max (mL/kg/min), predicted VO_2_max for normative body mass (mL/kg/min) and predicted VO_2_max adjusted for FFM (mL/kgFFM/min) for male and female.

Variable	All participants (*n* = 20)	Men (*n* = 3)	Women (*n* = 17)
Predicted VO_2_max expressed relative to total mass (mL/kg/min)	20.15 ± 5.00	24.09 ± 4.74	19.45 ± 4.84
Predicted VO_2_max for population normative adjusted body mass (mL/kg/min)	30.04 ± 6.58^b^	32.73 ± 2.02^a^	29.57 ± 7.02^b^
Predicted VO_2_max adjusted for FFM (mL/kg FFM/min)	38.55 ± 9.08^b^	41.89 ± 6.79^a^	37.96 ± 9.47^b^

*Note:* Data presented as Mean ± standard deviation.

Abbreviation: FFM, fat free mass.

^a^ *p* = < 0.05 (reference group is Predicted VO_2_max expressed relative to total mass [mL/kg/min]).

^b^ *p* = < 0.01 (reference group is Predicted VO_2_max expressed relative to total mass [mL/kg/min]).

**FIGURE 1:**
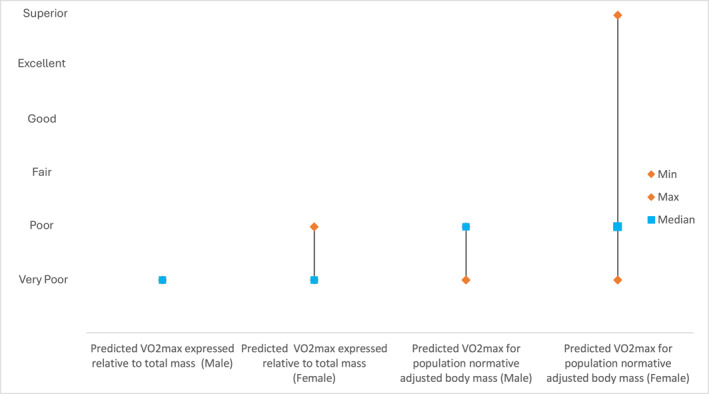
The median, minimum and maximum classification scores for the predicted VO_2_max for both male and female and the classification scores for the predicted VO_2_max when adjusted for population normative body mass for both male and female. The minimum and maximum classification score values are represented by the orange diamond, and the median classification score is represented by the blue square.

As seen in Figure 1, the median classification score for predicted VO_2_max for both male and female is 1.00 (very poor), whereas the median classification score for predicted VO_2_max when adjusted for population normative body mass for both male and female is 2.00 (poor). An increase of one classification category for predicted VO_2_max is seen when corrected for population normative body mass as compared to measured body mass. The maximum classification score increased from 2.00 (poor) to 6.00 (superior).

## 4 Discussion

This study aimed to explore how changing the expression of VO_2_max can influence the representation and interpretation of predicted VO_2_max in people that were awaiting MBS. Two approaches to adjust VO_2_ for body size without distorting the data due to excessive additional FM were explored. When body mass was estimated based on age and sex based normative BMI, and VO_2_ was expressed relative to this value, the median classification score increased from very poor to poor for both male and female and the maximum classification score increased from poor to superior within the female group. Anecdotally, this patient group typically has a range of physical capacity. Some are quite active, and activities of daily living can often be performed without undue cardiorespiratory distress. Therefore, the re‐classification better aligns with this experience.

Maximal oxygen consumption results are typically expressed relative to body mass, as more metabolically active tissue will use more oxygen at the same work output [18]. Maximal oxygen uptake results expressed in this manner also enable comparison between individuals across various body sizes [19]. Therefore, body mass has traditionally been used to take body size into consideration when VO_2_max is reported [20]. However, when there is a disproportionate amount of FM, this expression becomes distorted as the volume of lean mass, which is mainly responsible for increased metabolism during exercise, may not be accurately reflected [19]. The results of the study found that when predicted VO_2_max was expressed relative to total mass, VO_2_max (or the functional capacity of the cardiorespiratory system) was lower, when compared to both the correction for population normative adjusted body mass and for FFM.

Often predicted VO_2_max is used to determine functional performance and is therefore expressed relative to body mass as individuals must physically carry their mass around [21]. When functional performance—like walking up a hill or cycling a set distance—is determined using this measure, total mass is the most important parameter to consider. In addition, it is still important for overall body size to be considered as more active tissue will use more O_2_, but excessive amounts of relatively less active tissue like FM do not affect VO_2_ in the same manner [22]. However, when predicted VO_2_max is being used as a tool to evaluate the health and the integrity of the cardiovascular system prior to surgery, the metabolically active tissue (i.e., skeletal muscle) that is responsible for aerobic respiration should be more directly evaluated.

As patients undergoing MBS present with excessive FM, which is metabolically less active, their predicted VO_2_max results when expressed relative to total body mass may become misleading [23]. With this approach, the heavier the person is, the relatively lower their oxygen uptake will be and therefore their rating of aerobic capacity, regardless of their absolute VO_2_max or amount of metabolically active tissue during exercise [22, 23, 24, 25]. When predicted VO_2_max was expressed relative to total mass, this group all rated as very poor to poor fitness, and most would be deemed at increased risk of adverse events from surgery as a result. However, when their individually predicted VO_2_max was normalized to an average body mass, which discounted their excess FM, the average rating was more typical of an otherwise healthy but unconditioned person, with some in fact rating well. It has been observed previously that when predicted VO_2_max is expressed relative to body mass, the result is not often considered a reliable indicator of cardiorespiratory fitness in people that are awaiting MBS due to this compounding impact FM has on the reported VO_2_max result [23]. Therefore, if predicted VO_2_max is expressed relative to body mass and is being used as a tool to predict “fitness for surgery,” the outcome is possibly inaccurate unless alternative methods of adjustment are made, such as the ones explored within this study.

Examining predicted VO_2_max adjusted for population normative body mass enables the integrity of the cardiovascular system to be closely examined without the distortion of data due to excessive FM. This method for adjusting predicted VO_2_max accounted for body size by factoring their individual height to weight ratio but also assumes they have average fat mass [26].

Interestingly, within the female group, the maximum classification score increased from two (poor) to six (superior) when predicted VO_2_max was corrected for population normative body mass. If the higher values are more reflective of their aerobic capacity (by removing the influence of excess FM), then reporting predicted VO_2_max relative to total body mass, may be exaggerating the potential risk of adverse perioperative outcomes. Increased perceived surgical risk could lead to increased resourcing for an operation, resulting in an increased associated cost and burden, or may delay or prevent someone from accessing the surgery [27, 28].

Furthermore, research has indicated that lower cardiorespiratory fitness (VO_2_max < 15.80 mL/kg/min) is associated with longer operating times, incubation durations, estimated blood loss during surgery, and more frequent cardiovascular complications [3]. Within this study, two out of the 20 participants had a predicted VO_2_max (mL/kg/min) below this threshold, which were 12.70 and 9.29 mL/kg/min respectively. Therefore, these individuals would likely be excluded, even though the populations often utilized to determine these thresholds are elderly and present with a low to normal BMI, which is the opposite to that of populations that typically present for MBS [7, 12]. Hence, given the difference in population anthropometry and age, these thresholds may be altered if populations that are obese were utilized to determine these measures [29].

The ACSM has adapted the normative VO_2_max values used to classify predicted VO_2_max for age and gender from the Physical Fitness Assessments and Norms for Adults and Law Enforcement published in 2007 by the Cooper Institute [30]. Within each age group and gender classification of normative VO_2_max values (mL/kg/min) used for comparison within this study, a sample size of between 1,356 and 13,158 individuals was used to obtain these measures of VO_2_max. However, it is unclear whether these samples are representative of individuals with broadly different body compositions as body composition data were not made publicly available. Therefore, selection bias may be present in the reporting of the VO_2_max, given that people with greater BF% may be unlikely to participate in the assessments required to obtain predictions of VO_2_max. Hence, a systemic bias for higher normative VO_2_max measures for each age group and gender may be presented within the reported normative VO_2_max results. The impact of excessive FM on the representation of VO_2_max was further explored by adjusting predicted VO_2_max relative to FFM to examine the “physiological” ability of the cardio‐respiratory system to meet the oxidative demands of the metabolically active tissue within the body [24]. After predicted VO_2_max was normalized for FFM, individuals presenting for MBS appeared to have a similar predicted VO_2_max to that of individuals that are lean and sedentary, as reported in a previous study [31]. The study reported that when VO_2_max was expressed relative to FFM, there was no significant difference between fitness across two groups with broadly different body compositions (a group of individuals that are obese and a group of sedentary lean individuals) [31].

The lack of significant difference between these groups suggested that whilst body composition may differ between the groups given a significant difference between BF% was observed, the cardio‐respiratory systems ability to use oxygen (oxidative capacity) for aerobic respiration relative to their fat excluded body size may be similar. Therefore, these findings infer that individuals that are obese do not actually have a lower maximal aerobic capacity of their FFM, when compared to individuals that are lean [23]. Previous studies have indicated that when predicted VO_2_max is expressed relative to FFM, individuals with varying body composition have no difference in their predicted aerobic capacity [24]. Therefore, when predicted VO_2_max is normalized for FFM, it may provide a more accurate representation of fitness in people that are awaiting MBS. However, FFM includes bone mineral content, which does not contribute to aerobic respiration during exercise [23]. In addition, VO_2_max expressed relative to FFM cannot be compared to normative data or other reported thresholds that use total body mass, so finding suitable cutoffs for low fitness or increased risk will require greater effort [23].

It is important to note also that excess fat mass may play more of a role than simply arithmetic in oxygen uptake dynamics. The transportation of oxygen to the working skeletal muscle and the reduction in mitochondrial oxidation have both been found to be impaired by obesity [32]. Additionally, vascular function and the transportation of oxygenated blood during exercise may also be impacted by obesity [33]. Therefore, increased adiposity may subsequently reduce arterial‐venous oxygen content, ultimately reducing the predicted VO_2_max measures. The differences in predicted VO_2_max may also be explained due to the differences in gender split across the groups. The percentage of females within the group undergoing MBS was 0.15%, which compares to that of the group of obese sedentary individuals, which was 0.33% [31]. It has been well established that females, compared to their male counterparts, have a reduced VO_2_max due to the gender differences in physiology. That is, females often have a smaller heart, lungs, and hemoglobin mass when compared to males, which limits their capacity to deliver oxygen to the working muscle, ultimately reducing their VO_2_max, even when controlling for lean tissue [34].

Even though correcting for population normative body mass would enable the necessary FM to be taken into consideration, this correction is not specific to the individual and presents with potential sources of error dependant on the normative data being used for correction. For example, potential variables that would make the data less accurate include potential age ranges rather than specific ages and various inclusion and exclusion criteria of the participants selected within the population.

Therefore, correcting predicted VO_2_max for FFM enables VO_2_max to be corrected in an objective and individualized manner as FFM can be measured in a relatively simplistic method. Therefore, the development of normative VO_2_max data corrected for FFM would be beneficial for the categorization of “fitness” in people that are awaiting MBS.

This study is an opportunistic cross‐sectional analysis of real people who presented for MBS for weight loss and had both aerobic fitness and body composition assessed. It is limited by a small overall sample size and a particularly low number of males. These results are not intended to be generalizable, but rather to stimulate interest and analysis and prompt others to take up this question in a systematic prospective manner. In addition, further information in relation to the participants broader health status, adequacy of sleep and nutritional status was not collected. Hence, it is unclear how these factors may have potentially impacted the VO_2_max result achieved by the individual.

Normalizing VO_2_max relative to lean mass could be explored as a way of expressing “physical fitness” in people who present for MBS, given that the lean mass portion of body mass directly contributes to aerobic respiration [23]. Also, adjusting predicted VO_2_max for FFM or lean mass could be used as a way of screening for risk prior to MBS, with surgical complications explored as the outcome of interest.

## 5 Conclusion

This study reported two alternate methods for reporting VO_2_max, which suggested that expressing predicted VO_2_max results relative to total body mass may be underestimating aerobic capacity in people who are very obese, such as those undergoing MBS. Median classification scores increased from 1.00 (very poor) to 2.00 (poor) when predicted VO_2_max was expressed relative to population normative body mass. These findings confirm that more work is needed to understand the best way to present VO_2_ in people awaiting MBS.

## Author Contributions

Rebecca Dostan, Kade Davison, Belinda Durey, and Brett Tarca contributed to study design. Sara Slayman and Justin Bessell contributed to data collection. Rebecca Dostan performed the statistical analyses. Rebecca Dostan wrote the initial draft of the manuscript. Kade Davison, Brett Tarca, and Belinda Durey contributed to project administration and supervision. Authors provided final approval for the submitted version.

## Funding

The authors have nothing to report.

## Conflicts of Interest

The authors declare no conflicts of interest.
